# Geographical modelling of language decline

**DOI:** 10.1098/rsos.221045

**Published:** 2023-05-24

**Authors:** Douglas Brown, Steven Wrathmall

**Affiliations:** Department of Physics, Durham University, Durham DH1 3LE, UK

**Keywords:** complexity, simulation, language

## Abstract

Competition between languages affects the lives of people all over the globe, and a huge number of languages are at risk of extinction. In this work, statistical physics is applied to modelling the decline of one language in competition with another. A model from the literature is used and adapted to model the interactions among speakers in a population distribution over time, and is applied to historical data for Cornish and Welsh. Visual, geographical models show the simulated decline of the real languages studied, and a number of qualitative and quantitative features from the historical data are captured by the model. The applicability of the model to further real situations is discussed, as well as adaptations that would be needed to better take account of migration and population dynamics.

## 1. Introduction

Statistical physics has seen a remarkable number of applications to matters of our everyday lives. These include topics as diverse as the dynamics of traffic flow, stock market fluctuations, polarization in opinion polls and democratic elections, and the diffusion of ‘fake news’ on the Internet [1–7]. One additional topic where this approach has gained popularity is that of language, which is a fundamental part of the lives of almost everyone on the Earth. In recent decades, quantitative research into topics in language using tools from statistical physics and complex systems such as phase transitions, emergent behaviour and agent-based modelling has grown substantially [8–11].

Language decline is a particular area which has been studied in a number of ways from a quantitative perspective. Currently, there are approximately 7000 languages spoken in the world [12]. However, language decline is accelerating at an alarming rate, and over the course of the next century it is estimated that between 50% and 90% of these will become extinct [13]. Preserving languages is important from a number of perspectives. Language can be a defining aspect of many cultures, without which they would be homogenized as part of a larger group. Equally from a scientific perspective, languages can contain information on the history of a society and insights into the workings of the human brain [14,15].

Abrams and Strogatz were responsible for one of the first papers on the topic of modelling language decline, which motivates a simple differential model (henceforth referred to as the ‘ODE model’) by considering two languages, each of which has a certain status and is spoken by a certain fraction of the population [16]. Numerical solutions to the resulting parametrized equations are then fitted to empirical data for a range of real examples.

This model was comparatively simple, and a range of subsequent approaches have expanded upon this model theoretically, such as the inclusion of multilingualism, different interaction networks, population shift and reproduction and spatial dependence via reaction–diffusion equations [17–21]. Alternative approaches to a similar problem are also taken, such as using the Lokta–Volterra ‘predator–prey’ equations [22], the incorporation of more mechanistic details such as schooling and language in the home [23], and processed-based study of language diversity based on models commonly used in ecology [24].

However, there has been comparatively little further work in approaching the decline of real languages. Furthermore, while some of these models are ‘spatial’ in the sense of interaction networks or lattices [17,18], few consider concrete, real-world geographical features. One notable exception is the work of Burridge, where modelling of social interaction results in surface tension dynamics which is used to model the geographical distribution of real dialect boundaries [25,26]. These are applied to predict, with some accuracy, the positions of major dialect boundaries in the UK and Italy. Comparatively recently, the same author has considered the effects of migration patterns and social interaction on the geographical distribution of particular features of the English language, informed by data from dialectal surveys [27].

The approach of this work can be seen as complementary to the two models above. Where the former is used predominantly to model the equilibrium positions of dialect boundaries, and the latter is used to model the evolution of individual language *features*, the aim of this work is to adapt the former model to the evolution of boundaries between two competing languages. To the best of the authors’ knowledge, this is the first work that specifically models the spatial and geographical decline of real languages. The development of such a model could provide valuable insight to help minimize language extinction.

The two case studies in question examined in this work are those of Cornish and Welsh. These Celtic languages were both widely spoken in the British Isles for many hundreds of years, but both have suffered at the hand of English. Welsh experienced a substantial decline in usage during the nineteenth and twentieth centuries, and despite recent efforts by the Welsh government, the future of the language is by no means certain [28,29]. Cornish experienced a similar decline over the course of the last millennium and was finally declared extinct at the end of the eighteenth century, although it has experienced recent revival efforts [28]. The data used in this research comes from historical estimates of the number of speakers using church records in the case of Wales [30], and evidence from contemporary writing and settlement names in the case of Cornwall [28,31]. However, in contemporary Britain, use of minority languages is now recorded in census data [32,33]. Similar data are available for many other countries, and the Council of Europe has a Charter for Regional or Minority Languages, which promotes the usage and study of minority languages in Europe [34].

The decline of these languages is, of course, a complex issue containing myriad social, economic and political factors. However, the aforementioned literature makes it clear that much can be done with a minimal model. In this case, the geography, the population distribution and the way this population interacts is used to model the competition between the two languages and the decline that takes place.

The model on which this work is based and the adaptations made to it are outlined in §2, and the method of applying this model and the comparison to real data is described in §3. Results and discussion are then presented in §§4 and 5, respectively.

## 2. Theory

In this section, we briefly illustrate the model first developed in [25] by Burridge. More detail can be found in the original work.

In order to model a population of speakers, consider a population density, *ρ*(***r***). An infinitesimal region of this population, *δA* (***r***_1_), centred on position ***r***_1_ contains a certain number of speakers. These speakers interact with those closer to them more than they do with those further away. Equally, a speaker in a more densely populated region will have more interactions in a given timeframe than one in a less populated region.

This leads to an interaction density, *ψ*, between speakers in two locations, ***r***_1_ and ***r***_2_:

2.1 $$\psi (r_{1},r_{2})\;\delta A\;:=\left[\frac{1}{\exp(d_{12}^{2}/(2\sigma^{2}))}\frac{\rho (r_{2})}{N(r_{1})}\right].$$

Here, *d*_12_ is the straight-line distance between positions ***r***_1_ and ***r***_2_, N is a normalization constant so that $\int_{R^{2}}\psi \;\;dr_{2}=1$, and *σ* controls the length scales of interactions.

We consider speakers to have a choice of two languages and model the frequency of use of each. Ignoring bilingualism, for two languages *A* and *B*, the frequency of usage of language *A* at time *t* and location ***r*** is *f*_*A*_(***r***, *t*) = 1 − *f*_*B*_(***r***, *t*). Even in the case of bilingualism, we can consider *f*_*A*_ and *f*_*B*_ to be the usage of the languages as the *primary* language of interaction.

The mean frequency with which a language is used is given by the spatial average of *f*(***r***), weighted by the interaction density:

2.2 $$\bar{\;f}(r,t)=\int_{R^{2}}f\;(r^{\prime },t)\psi (r,r^{\prime })\;dr^{\prime }.$$

A time dependence can then be added by using the so-called ‘forgetting curve’, whereby recollection ability declines exponentially over time [35]. This is not intended to be a psychologically accurate model of how the brain acquires and loses language, simply a practical way to model the fact that more recent interactions will have more of an effect on how a speaker uses language at the current time. This is done by defining a speaker’s memory, *m*, as:

2.3 $$m(r,t)\;:=\int_{-\infty }^{t}\frac{1}{\tau }\exp\frac{\left(s-t\right)}{\tau }\;\bar{\;f}(r,s)\;ds,$$

where *τ* controls the timescale over which interactions are forgotten.

The final puzzle piece is the relationship between a speaker’s current use of language and their memory of use in the past. Human beings are known to have a strong tendency to conform to others in their social environment [36], which can be taken account of mathematically by using a sigmoid function to fix the relationship between *f* and *m*:

2.4 $$f=\frac{m^{\alpha \beta }}{m^{\alpha \beta }+{\left(1-m^{\alpha }\right)}^{\beta }}.$$

The parameters *α* and *β* control, respectively, bias for or against a particular language (higher *α* means more pressure to conform to that language in particular) and the pressure speakers feel to conform to others around them (regardless of which language is spoken). Appendix A shows a plot of equation (2.4) and the effects of *α* and *β*.

A differential equation which can be used to model the evolution of languages can be arrived at by simply differentiating equation (2.3) with respect to time and rescaling by *τ*:

2.5 $$\frac{\partial m(r,t)}{\partial t}=\bar{\;f}(r,t)-m(r,t).$$

This can be simplified using the saddle point method, as discussed in [25]. Assuming that $\nabla^{2}\rho \ll \sigma^{2}$ removes the dependence of equation (2.5) on the explicit integral for $\bar{\;f}$, leaving:

2.6 $$\frac{\partial m(r,t)}{\partial t}=f\;(r,t)-m(r,t)+\frac{\sigma^{2}}{2}\frac{\nabla^{2}(\rho (r)\cdot f\;(r))}{\rho (r)}.$$

Hitherto the model described is as motivated by Burridge. However, one aspect of this equation is that the rate of change of language use is dependent only on the population *gradient*, not on the population magnitude itself. In Burridge’s work, this is a useful feature which is successfully exploited in finding equilibrium positions of dialect boundaries. However, when explicitly considering the time evolution of language decline, this has the result that use of a language declines at an equal rate in areas with different population densities. Both intuition and the fact that minority languages are more commonly spoken in rural or isolated areas makes this an undesirable feature of the model [37]. In fact, there is nothing in this model that specifically pertains to language—we could equally be considering any other form of tradition, culture or social behaviour.

To counteract this feature of the model, a simple alteration is made so that the interaction range is now dependent on the population density, via:

2.7 $$\sigma (\rho )\;:=\sigma_{0}(1-e^{-k\rho }),$$

with *σ*_0_ representing the maximum interaction range. This function is chosen arbitrarily, but has useful properties: when the population is zero, *σ*(0) = 0, and the function increases smoothly and asymptotically reaches *σ*_0_, scaled by a factor *k*. The high-*k* limit corresponds to constant *σ*. The effect of such a function on the results of simulation is shown in appendix B.

In the case studied here, we can expect the net result of this to be twofold. Firstly, rural areas will hold on to their language more so than they would with a single static interaction range, and the effect of cities ‘drawing in’ speakers from further afield will be exacerbated further. This aligns with recent work showing that increased road density and easily navigable terrain are both correlated with higher rates of language endangerment [38].

## 3. Method

The above differential equation (equation (2.6)) is solved numerically using the finite difference method [39]. This involves initializing the various scalar fields (*ρ*, *m*, *f*) on a discrete grid, and numerically calculating a discrete Δ*m* which is to be multiplied by a chosen timestep Δ*t*.

An important part of this work is the real data used as comparison. Although this work looks at the long-term evolution of language boundaries, historical population data before mass censuses is difficult to obtain. This work makes the same assumption as Burridge that a modern population distribution can be used as a proxy for the past. Most major UK settlements have been in existence for centuries (indeed, most in Cornwall since at least the thirteenth century) and an attempt to model the relative historical population distribution more accurately would be complex enough and require enough assumptions to deserve an investigation of its own. The approach of this work is to see what can be done with a model of minimal complexity, and the results are evaluated in the context of the assumptions made.

Recent population data and outlines of geographical regions are available from the UK government. This work uses the census Output Areas used by the Office for National Statistics, which have a recommended sizes of 125 households [40–42]. In order to create a smooth population distribution from the census data, the population spike at each grid location is replaced by a Gaussian distribution, with population falling beyond the coastline redistributed iteratively. The standard deviation of this spread—referred to as the smoothing radius, *σ*_*s*_—is somewhat smaller than the interaction range *σ*.

Historical estimates are available for the Cornish speaking area and population over the course of the last thousand years [28,31]. These are obtained using a mixture of writings from the time and evidence from place names. While the data for the Cornish-speaking population is given back to the year 1000 CE, the geographical area covered is only estimated after 1200 CE. At this point, the River Tamar and the River Ottery are said to be the boundary of the Cornish-speaking region [28]. Since the geography of the system is an important factor in this model, this condition is a useful starting point. It is well beyond the scope of this work to estimate the uncertainty in this historical data, but it will provide useful comparison to evaluate the predictions of the model.

Data on the usage of Welsh over the time period considered is more detailed, and reasonably reliable initial conditions can be found. One source uses church records from the nineteenth century to divide Wales into regions favouring either English or Welsh, or those that were bilingual [30]. The borders between the bilingual region and the region favouring English were chosen as the initial condition for the simulations. For reasons of simplicity, it was decided not to attempt to align the transition region that forms in the model with the bilingual region in the historical data.

As introduced in §2, there are a range of parameters which must be considered, and inevitably some of these will be estimates. Furthermore, owing to the intensive nature of the simulations, it is impractical to consider variations of all possible parameters. Given that this work is considering linguistic decline, it seems logical to place emphasis on *α*, the bias parameter. Previous work shows that equilibrium boundaries can form when a population distribution is considered, but this is precisely not the case here, so varying the bias parameter beyond equilibrium will be of key importance [26].

Two further important parameters are the conformity, *β*, and the interaction range, *σ*. These both influence the width, *w*, of a transition region between two linguistic variables via *w* ≈ 1.8*σ*(*β* − 1)^−1/2^, as shown in previous work [25]. A larger interaction range intuitively brings about a wider transition region. The effect of a high conformity can be seen in the limit *β* → ∞ where the function in equation (2.4) becomes a step function, and so the transition region vanishes.

In previous work, the interaction range is estimated to be of the order of 10 km using average travel times to work and school. The approximate width of a known dialect transition region in the UK is then used to deduce a value for *β* of 1.1. However, in the periods considered—before the age of mass transport—it seems unlikely that people would regularly find themselves travelling as far as 10 km. Indeed, estimates for the area of medieval UK cities suggest that in the fourteenth century, London—the largest by far—had an area of 3.3 km^2^, giving an approximate diameter of 2 km [43]. It is, of course, an approximation to assume a static interaction range throughout the centuries, but a value of 5 km was chosen here. For similar reasons, the metropolitan interactions considered in [26] are not considered.

A further matter for consideration is how to map the simulated decline to real years. For Cornwall, since the ‘end result’ is known we have the boundary condition that the number of speakers should reach 0 (although in reality a small threshold is used) in the year 1800 CE. Simulations that result in decline can therefore be scaled between these two endpoints. The situation with Welsh is more complicated and the language is, of course, very much alive. An initial point is used, as are a selection of historical data points up to (almost) the present day. In this case, simulations are scaled so as to minimize the mean squared error (MSE) between the historical points and the simulations.

## 4. Results

Two different and contrasting case studies were chosen: the decline and extinction of Cornish in Cornwall over the course of the second millennium CE, and the decline of Welsh in Wales from the 1800s until the present day.

### 4.1. Cornish

**Figure 1.**
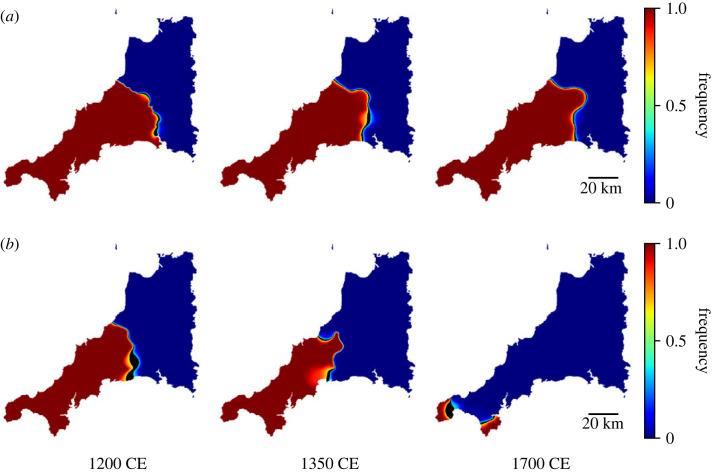
Figure showing simulated decline of Cornish for different values of *α*. The colour shows the frequency of use of Cornish by speakers in that location. The black line shows the points where *f* = 0.5. The scale is shown in the right of the figure. (*a*) A simulation with insufficient bias (*α* = 1.0) results in a stable dialect boundary with no decline. (*b*) Simulation of Cornish with *α* = 1.5 at years 1200, 1350 and 1700 CE.

Figure 1 shows a simulated decline of Cornish for two different parameter sets. The frequency of use of Cornish is shown in colour, and the black line shows the line of 0.5 frequency (with a small tolerance either side). With neutral bias—*α* = 1.0, shown in figure 1*a*—a stable language boundary forms, with the English boundary held back by the effect of the population gradient. Increasing the value of *α* eventually overcomes this effect, and causes Cornish to decline further. For sufficiently high values of *α*, the only stable configuration of the system is where Cornish is no longer spoken, seen in figure 1*b*. Clearly, this is the set of scenarios of interest, given that we know the history of the study in question.

**Figure 2.**
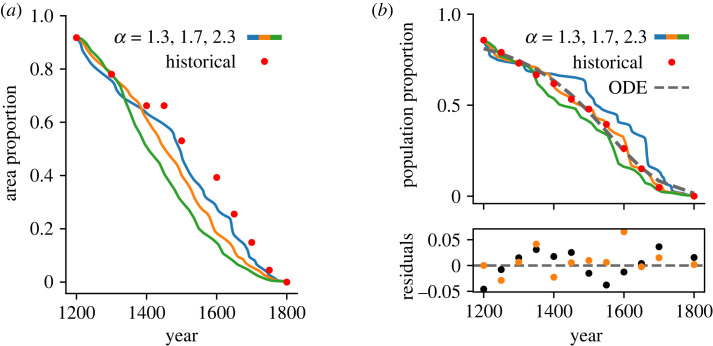
Figure showing the Cornish-speaking area and population over time. Coloured lines show the simulation data with *α* ranging from 1.3 (blue) to 2.3 (green). Other parameters are: *β* = 1.1, *σ*_*s*_ = 2 km, *σ*_0_ = 5 km, *k* = ln2/10. (*a*) shows the Cornish-speaking area over time for different values of *α*; and (*b*) shows the Cornish-speaking population over time for different values of *α*. Simulation data are vertically scaled to match the initial condition. The ODE model’s solution is shown in black. Residuals are shown for the ODE model (black) and for *α* = 1.7 (orange).

For simulations where Cornish *does* eventually decline, the Cornish-speaking area and population are shown in figure 2. The initial and final points in the simulation are scaled so that they match the endpoints of the historical data—1200 and 1800 CE, respectively. Figure 2*a* shows the modelled Cornish speaking area as a proportion of the area of the county for a range of values of *α* (shown in colour), with red points showing the historical data. Figure 2*b* shows the modelled population proportion (coloured lines), the historical data (red points) as well as the optimized curve from the model discussed in [16]. The lower plot shows the residuals for this model and for the best-fitting of the simulations (with *α* = 1.7) as described by the MSE with respect to the historical data.

The initial population fraction in the simulated data appears to be different to that of the historical data. The initial condition for the simulation was set according to the paths of the Rivers Tamar and Ottery, as mentioned previously and discussed in [28]. The initial population fraction for the model is then found by taking the population distribution of this initial Cornish-speaking area and dividing by the estimated total population at the time. The coincidence of the initial simulated and historical *areas* suggests that the initial condition itself is not to blame. As an illustration, the initial region used in the model covers 96% of the area of the modern county of Cornwall and contains 95% of its population. The historical data estimates the Cornish-speaking region in 1200 CE to cover 92% of the area, but contain an estimated 86% of the population. A skew in the population distribution towards an area not included in the initial region may result in the situation shown. Other possible explanations seem less likely. While minor expansion beyond the initial condition does occur, the coincidence of the initial areas suggests this is not responsible for the offset seen here. Equally, the boundary between Devon and Cornwall has been the River Tamar since at least 936 CE, so a systematic error in using the total county population is unlikely too. It is also possible that the population estimates are inaccurate, but it is not the place of this work to speculate on such. In the case where a skewed population distribution is causing this offset, it is reasonable to rescale the population decline so that the simulation is using the correct initial condition. It is this rescaled data that is shown by the coloured lines in figure 2*b*, which is justified by the above. The full original data are shown in appendix C.

The dashed line in figure 2*b* shows the solution to the ODE model [16] for the historical data. This appears to fit the data well (with an MSE the same as that of the best-fitting simulation) but it is clear in the residuals (shown in black) that this model is systematically over- and under-estimating the historical speaker population owing to the simple, analytical nature of the function. This is, of course, a different approach to the one taken by this work: fitting a curve *post hoc* to all the historical data points, as opposed to using only the start and end points, and allowing the simulation to evolve between the two. The residuals for the best simulation are shown in orange, and do not show a discernible trend. The one particularly high residual is perhaps better interpreted as a minor horizontal displacement, rather than the more extreme vertical discrepancy that it appears at first to be.

**Figure 3.**
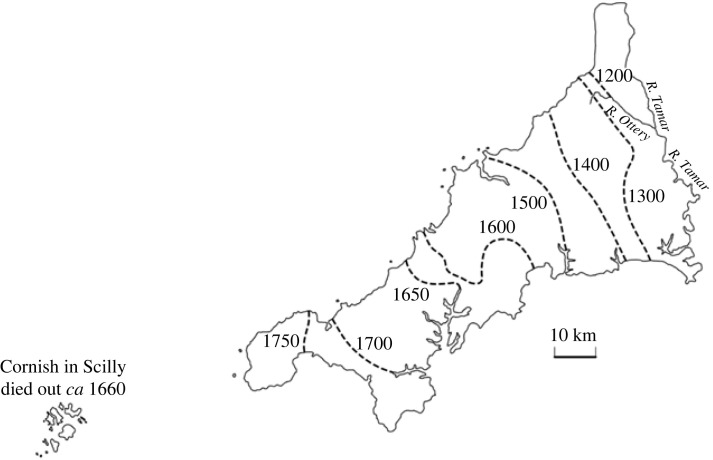
Westward retreat of traditional Cornish (reproduced with permission from [28]).

As a further point of comparison, figure 3 shows a reproduction of the estimated timeline of the geographical decline of Cornish as estimated by linguists (reproduced with the publisher’s permission from [28]). A number of the qualitative features in this map are reproduced in the simulations, including the slight eastwards ‘bulge’ around 1300 (shown in 1350 in the simulations), and the final retreat of the language to the western ‘toe’ of the peninsula. The first feature may be owing to Bodmin moor, a remote area in central western Cornwall. If so, this is a prime case of the importance of population density on the movement of the boundaries.

Finally, it is insightful to briefly comment on a variation of this simulation using the same model but where the population distribution is flat, i.e. *ρ*(***x***) = 1. The initial condition used is the same. In this case, no stable boundary forms, even for neutral bias (*α* = 1.0). This suggests that Cornish—before considerations like bias or population distribution—was already at a geographical disadvantage. The non-parallel north and south coastlines of the county mean that, in the absence of a population distribution to hold back the encroaching boundary, the surface tension property of the language boundary inevitably causes shrinking of the Cornish-speaking region.

### 4.2. Welsh

**Figure 4.**
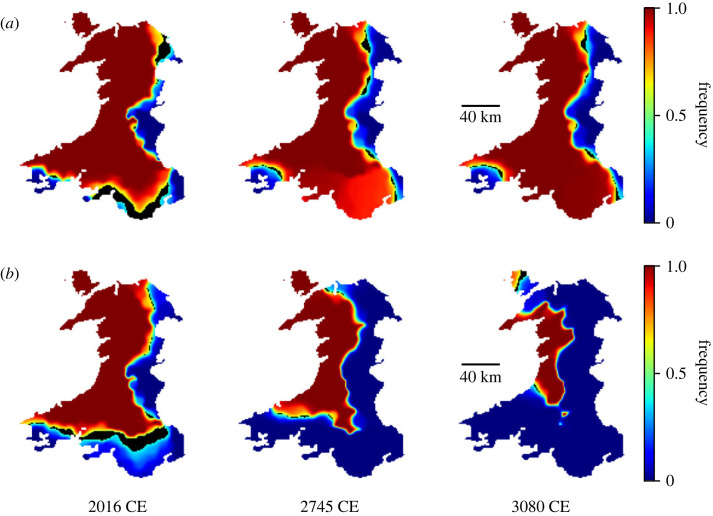
Figure showing simulated decline of Welsh for different values of *α*. The frequency of use of Welsh is shown by the colour, and the black line shows the regions where *f* = 0.5. The scale is shown to the right of the figure. (*a*) A simulation with insufficient bias (*α* = 1.0) results in a stable dialect boundary with no decline. (*b*) Simulation of Welsh with *α* = 1.7 at years 2016, 2745 and 3080 CE.

The simulations of Welsh are shown in figure 4. As with Cornish, figure 4*a* shows a stable boundary forming (along an interesting possible alternative English–Welsh border, along the Brecon Beacons and the Cambrian mountains), and an example of decline is shown in figure 4*b*.

This example is somewhat less trivial than that of Cornwall. The triangular nature of the Cornish peninsular means that there is really only one direction for decline to progress. Despite the more complicated geography here, a number of qualitative features of the decline are accurately predicted: rapid decline westwards along the north coast, small areas left in the lower centre, and the most persistent areas of Welsh in Angelsey and the Ll$\hat{y}$n peninsular in the northwest.

**Figure 5.**
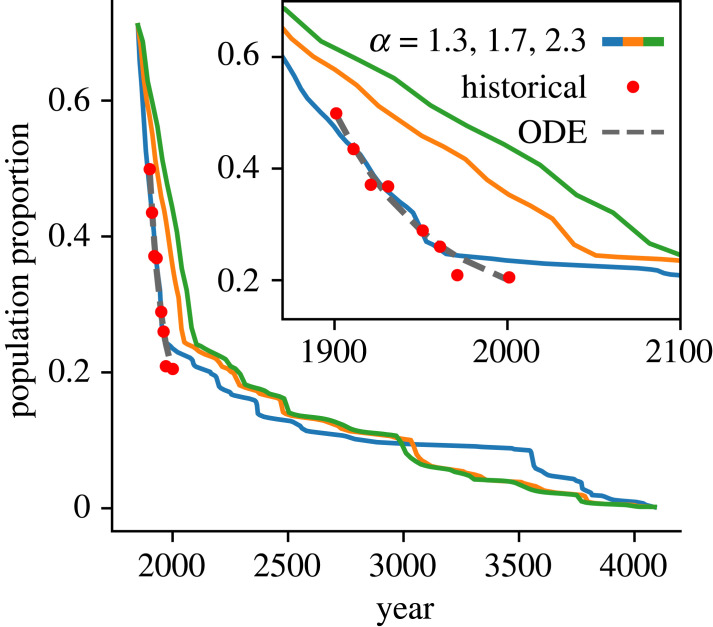
Figure showing decline of the Welsh speaking population. Coloured lines show the simulation data with *α* ranging from 1.2 (blue) to 2.3 (green). The *x*-axis scaling has been calculated by optimising MSE values with respect to the historical data (red points). The black line shows the optimized differential equation model (ODE). Other parameters are: *β* = 1.1, *σ*_*s*_ = 2 km, *σ*_0_ = 10 km.

Figure 5 shows the predicted decline of Welsh according to different simulations, along with historical data and the prediction of the ODE model. Of the values of *α* tested, *α* = 1.3 fitted the best, with a comparable MSE to that of the ODE model. The *x*-axis was scaled by minimizing the MSE between the historical points and the simulations. The prediction of the model that Welsh will eventually become extinct around the year 4000 CE should of course be taken with a pinch of salt, since languages themselves change dramatically over such timescales. However, the broad prediction is that the recent steep decline will soon reach a slower, more gradual decline, with the *α* = 1.3 showing a quasi-plateau.

**Figure 6.**
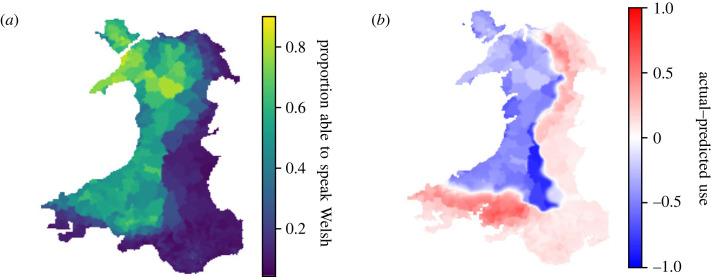
Figure showing current usage of Welsh according to census data (*a*) and comparison to predictions (*b*). (*a*) shows current proportion of those in Wales able to speak Welsh. Source: Welsh Government [32]. (*b*) Map showing difference between actual and predicted usage, using census data from (*a*) and timestep with lowest total mean squared difference for *α* = 2.3.

The UK census, taking place every 10 years, records the number of Welsh speakers, and much of these data are made available [32]. This means that, in contrast with the Cornish case study, there is detailed, geographical data available on the number of Welsh speakers in the country—these data are shown in figure 6*a*.

The snapshot in figure 6*b* shows the difference between the actual and predicted usage at the time step which has the lowest MSE from the 2011 census data, over all values of *α* examined. Red shows regions where the actual proportion of speakers is greater than that predicted, and blue shows lower. Since the model characterizes a Welsh speaking area as having a frequency of 1.0, and the highest output area has a Welsh speaking proportion of only 91%, clearly most of the map is expected to be blue. The particular features which stand out in this case are the Brecon Beacon mountains in southeastern Wales—where the Welsh speaking population is overestimated—and the very northern coast—where the decline at this point is accurately captured.

In the case of the Brecon Beacons, this may be an area where the dependence of the interaction range on the population density may be overly strong; clearly, the language boundary is being clung onto by the lower population density in that region.

The availability of these data means that there is now a second set of real data available to add a timeframe to the simulations. The time step where the difference between the simulated and the actual proportion of Welsh speakers is minimized can be considered the frame best representing the year 2011. Assuming a linear time scale, this can be extrapolated to the ‘end point’ where the usage will (supposedly) reach 0. Using this method, the timescale is significantly shortened, with a predicted extinction date of approximately 2600 for *α* = 2.3. The qualitative shape of the predicted decline is, of course, the same, with an extended plateau for much of the time period.

## 5. Discussion

Sufficient bias against one language in a two-language system causes decline to take place in this model. However, despite the intentions to keep this model relatively minimal, there are still a number of other parameters which could have their effects examined in more detail.

For example, the transition region in the simulations is rather narrower than historical estimates may suggest [28]. Decline happens in the appropriate regions at appropriate rates but clearly the current transition region is too binary. Changing the conformity (*β*), possibly dynamically with frequency of use of a language, would affect the width of the transition region as discussed previously, and may lead to the intuitive effect that people’s preferences for language change with the circumstances around them. Since the simulations are very intensive a brute-force search is perhaps not suitable, but machine learning could be used to optimize within the parameter space.

The scaling of the predicted population decline to the historical data and the spatial analysis over the whole region provide rather different long-term predictions in this case. Where the data are available, the latter method is perhaps less crude, with many more data points used in the comparison, and is less susceptible to uncertainties in the population distribution used, as seen in the Cornwall case study.

Another important issue is that of migration. For example, the initial condition used for Wales shows English favoured in parts of the south despite the rest of the country being Welsh-speaking. The reason for this is owing in part to the Anglo-Norman invasion of Wales shortly after 1066, and partly owing to migration into the mining areas of south Wales during the industrial revolution [44]. Large-scale human migration could not be accounted for by this model.

This simplification is probably of less concern for the Cornish simulations owing to the lack of mass transport during any period of the language’s prevalence. However, migration probably plays a much more important role in the Welsh case study, and this is something which should be considered in future work.

On a related note, the model here also does not take into account the inhomogeneous nature of Welsh-speaking regions. Much of the north of the country has an average frequency in the region of 60–80%, and even the most Welsh-speaking region (near Caernarfon in northern Gwynedd) has a frequency of only 90%.

Internal destruction of a dialect—including by migration—is a phenomenon considered in [26], and may be a factor in play here. A simple model shows that inward migration of speakers of one variant into an area where another variant is dominant can cause erosion of a community of speakers from the inside, rather than by the movement of an isogloss.

The approximation of a static population distribution was motivated by the nature of the time periods being considered, but may not withstand the effects generated by widespread long-distance travel, now commonplace in developed countries. As early as 1850, small ‘islands’ of bilingualism can be seen in majority Welsh-speaking regions [30]. These regions are not able to arise naturally in the local-only model considered here, and their influence on the linguistic geography of the surrounding region is probably underestimated here. However, introducing the so-called ‘metropolitan’ areas motivated by Burridge in [26] is not straightforward and leaves a lot of room for ambiguity in the model (such as definition of what constitutes such an area and how that may change over time), not to mention further computational overhead.

A further assumption has been made that the current population distribution is a suitable proxy for those in the past. This was made because past population data before the era of mass census is difficult to obtain and rely on, and was justified by the remark that most population centres in the UK have been in existence for centuries. However, data show that different types of regions have experienced different rates of population growth. For example, the city of Cardiff has grown in population by a factor of more than 50 since the year 1801, but the total population of Wales has increased by only a factor of 5 in the same period [44]. The relative gradient between rural and urban areas should therefore increase over the time period examined. For applications of this model in the modern era, it may be pertinent to incorporate a dynamic population distribution using census data and population projections.

It is also worth noting that social and political action can affect the state of a minority language. For example, the Welsh government is aiming to reach 1 million Welsh speakers by 2050 and the improvement in Welsh and bilingual schools has helped to increase the number of children aged 3–4 who can speak Welsh from 18.8% in 1971 to 23.6% in 2011 [29,45]. This model is not currently well-adapted to account for intergenerational change in language use, but future work could take inspiration from the Welsh government’s projections for the number of Welsh language speakers, which takes into account the differing use of language with age [45].

## 6. Conclusion

The aim of this work has been to examine whether, and to what extent, the statistical physics model developed in [25,26] to model stable dialect boundaries can be used to model the decline of minority languages. This work is something of a proof of concept, being (to the best of the authors’ knowledge) the first geographical model of real language decline. Simulations of population decline against historical data are shown to have comparable accuracy to the ODE model of Abrams & Strogatz [16], but crucially this model is also able to produce geographical simulations of language decline, which reproduce certain qualitative features of real language decline. Furthermore, the model only relies on an initial condition, where previous models have fitted trends *post hoc* over a whole historical period. This means that, in principle, all one needs to apply the model is a single snapshot of population distribution and language use. This model could be applied to identify regions where minority languages are most at risk, and so help to inform policy and educational practices.

From a linguistic perspective, it is worth noting how much of the decline of the languages studied seems to be possible using only geographical features, without knowledge of any historical, social or political events occurring at the time. In the case of Cornish, events such as the Prayer Book Rebellion and the Cornish revolutions are often seen as being partly responsible for its decline around the middle of the last millennium [28,46]. The work here suggests, however, that the geography of the region in question may play a greater role than is typically imagined.

The application of this model to other languages requires only geographical data for the region of interest and an initial condition for language use, both of which are typically available from census data. As discussed, the model as-is is currently best-suited to regions without regular, substantial movement of speakers between population centres, which of course excludes much of the industrialized world.

Recent work has shown that the road density and more navigable terrain both correlate with greater language endangerment [38]. Accurate modelling of decline in these areas should, of course, include these factors. However, the current model could nonetheless be used to see which areas are more geographically vulnerable to decline, and to model which parts of the world’s less industrialized regions are most at risk of language decline, which could then inform specific policy points as in [23].

It is also worth re-emphasizing that there is nothing in this model that specifically pertains to language. The interactions within a population here could just as easily be used to track and predict the changes of any other features of human culture which can reasonably be modelled by this simple model of interactions between members of a population.

## Figures and Tables

**Figure 7.**
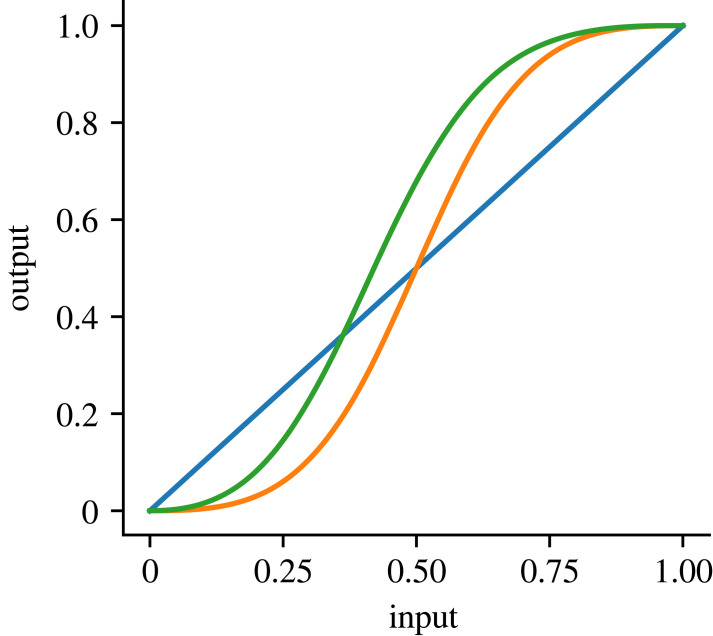
Illustration of the sigmoidal relationship given to current usage frequency and memory, equation (2.4), for different values of *α* and *β*. The blue line (*α* = *β* = 1) illustrates no conformity or bias. The orange line (*α* = 1, *β* > 1) illustrates conformity, while the green line (*α* < 1, *β* > 1) illustrates both conformity as well as bias towards a favoured variant.

**Figure 8.**
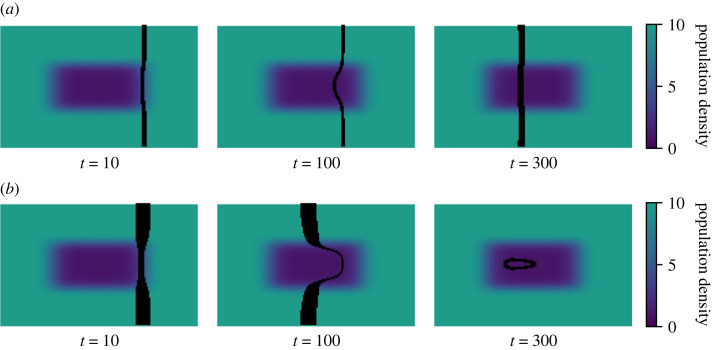
Figure showing language boundaries for a system with a uniform background population with a central sparsely populated region at *t* = 10, 100, 300 in arbitrary time units. Two variants are present in the system, with the preferred variant encroaching from the right. The boundary between the variants (*f* = 0.5 ± 0.2) is shown in black. Other parameters are: *α* = 1.05, *β* = 1.1. (*a*) *σ* = 1.0. (*b*) *σ* = *ρ*(***r***)/2.

**Figure 9.**
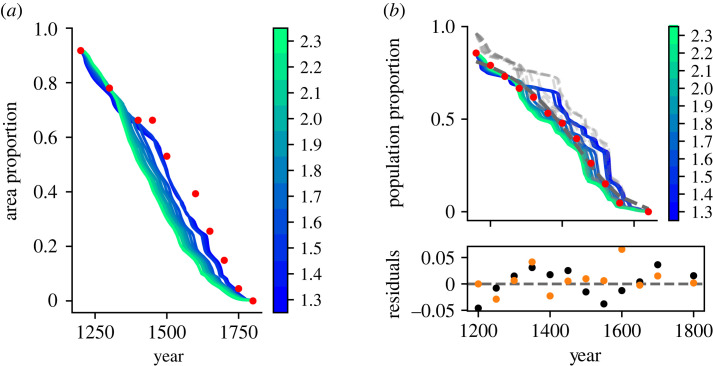
Figure showing the Cornish-speaking area and population over time. Coloured lines show the simulation data with *α* ranging from 1.3 to 2.3. Other parameters are: *β* = 1.1, *σ*_*s*_ = 2 km, *σ*_0_ = 5 km. (*a*) shows Cornish-speaking area over time; and (*b*) shows Cornish-speaking population over time. Scaled data are shown in colour with original data in grey. The ODE model’s solution is shown in dark grey. Residuals are shown for the ODE model (black) and for *α* = 1.7 (orange).

## Data Availability

Data is available from the Dryad Digital Repository: https://doi.org/10.5061/dryad.fn2z34txq [47].

## Appendix A. Relationship between memory and current frequency

The function motivated by Burridge [25,26] to represent the relationship between a speaker’s past experience and their current use of language is the sigmoidal function in equation (2.4), reproduced here:

$$f=\frac{m^{\alpha \beta }}{m^{\alpha \beta }+{\left(1-m^{\alpha }\right)}^{\beta }}.$$

Here, *α* represents the bias for or against a particular language variant and *β* represents the pressure on a speaker to conform to language around them. As shown in figure 7, a change from *β* = 1 (blue) to *β* > 1 (orange) causes the input (past experience) to disproportionately affect the output (current usage). As a crude example using the illustrated graph, for a particular binary feature of language, a speaker need only hear a particular feature 75% of the time to use it almost 100% of the time—i.e. there is pressure to conform.

The orange line has no bias for or against a particular variant (*α* = 1). On the other hand, the green line shows a situation where *α* < 1, which causes bias in favour of the particular variant being measured. Here, a speaker hearing a variant 50% of the time would be pushed towards using it more than 50% of the time owing to the bias towards that feature. This models the situation where one variant (such as use of a particular word or vowel sound, but this can be a proxy for a language or dialect as a whole) is more preferable to speakers in that region, be it for social, economic or cultural reasons.

## Appendix B. Motivation for variable interaction range

Burridge’s model is designed to find stable dialect boundaries. Forces only depend on gradients, not on absolute values. For our purposes though, this means that boundaries move with equal speed regardless of population density. We would expect the rate of language change to be smaller in rural or sparsely populated areas, hence the motivation for a population-dependent term in equation (2.7).

Figure 8 illustrates this effect. An idealized population distribution is chosen which is uniform except for a sparsely populated region in the centre (shown in purple). This could represent a geographical feature such as a hill or moor. Two variants are initialized, with the preferred variant encroaching from the right. Figure 8*a* shows the evolution for a uniform value of the interaction parameter *σ* = 1.0, and figure 8*b* shows evolution for *σ* = *ρ*(***r***)/2.

For constant *σ*, the boundary between the variants moves at an approximately constant rate through the frame, and remains essentially vertical. The evolution for variable *σ* shows a slower uptake of the new variant in the more sparsely populated region, with a small ‘island’ of use of the old variant in the sparser region at *t* = 300 while the new variant is dominant in the whole surrounding area.

For the purposes of this toy illustration, a simple dependence *σ* ∝ *ρ*(***r***) was chosen. Given that the real population distributions under consideration are on a much larger scale than those here, a more nuanced relationship than simple direct proportion is needed. The function chosen as an ansatz is:

B1 $$\sigma (\rho )\;:=\sigma_{0}(1-e^{k\rho }),$$

with *σ*_0_ the maximum interaction range. With no population in an area, *σ*(*ρ*) = 0, clearly, and since a null population has no gradient, the last term of equation (2.6) is 0 anyway. The function increases smoothly and asymptotically approaches *σ*_0_, at a rate scaled by *k*. The high-*k* limit corresponds to constant *σ*.

## Appendix C. Scaling of population data for Cornwall

As discussed in §4, the initial Cornish-speaking population in simulations did not align with the historical data, despite the historical region being used. For the reasons explained previously, it is likely that an imbalance in the population distribution is responsible for this, and the simulation was rescaled to coincide with the correct initial condition. (This was the only way in which the simulation data was altered.) For the sake of completeness, the data are replicated below with the original unscaled data shown in grey in figure 9.
